# Performance evaluation of medical service for breast cancer patients based on diagnosis related groups

**DOI:** 10.1186/s12913-021-06439-8

**Published:** 2021-05-24

**Authors:** Xinkui Liu, Furong Liu, Lin Wang, MengFan Wu, LinPeng Yang, Le Wei

**Affiliations:** 1 Department of Medical Records Management, The First Affiliated Hospital of Zhengzhou University, 1# Jian She Dong Road, Er Qi Distict, Zhenzhou, China; 2 Radiotherapy Inpatient Ward II, The First Affiliated Hospital of Zhengzhou University, 1# Jian She Dong Road, Er Qi Distict, Zhenzhou, China

**Keywords:** Diagnosis related groups (DRGs), Medical services, Breast cancer patients, Performance evaluation

## Abstract

**Background:**

To evaluate the performance of medical service for patients with breast cancer in Henan Province, China, using diagnosis related groups (DRGs) indicators and to provide data to inform practices and policies for the prevention and control of breast cancer.

**Methods:**

The data were collected from the front pages of medical records (FPMR) of all hospitals above class II that admitted breast cancer patients in Henan Province between 2016 and 2019. Breast cancer patients were the subjects in our study. China DRGs (CN-DRGs) was used as a risk adjustment tool. Three indicators, including the case mix index (CMI), number of DRGs, and total weight, were used to evaluate the range of available services for patients with breast cancer, while indicators including the charge efficiency index (CEI), time efficiency index (TEI) and inpatient mortality of low-risk group cases (IMLRG) were used to evaluate medical service efficiency and medical safety.

**Results:**

Between 2016 and 2019, there were 103,760 patients with breast cancer. The total weight increased over the study period at an average annual rate of 21.71%. The TEI decreased over the study period by 15.60%. The CEI exhibited an increasing trend, but the average annual rate of increase was small (2.94%). The IMLRP was 0.02, 0, 0 and 0.01% in 2016, 2017, 2018 and 2019, respectively.

**Conclusion:**

The performance of medical service improved between 2016 and 2019 for breast cancer patients discharged from study hospitals in Henan Province. The main area of improvement was in the range of available services, but medical institutions must still make efforts to improve the efficiency of medical services and ensure medical safety. DRGs is an effective evaluation tool.

## Background

Breast cancer is the most common malignancy in women, and approximately 11% of breast cancer cases worldwide occur in China [1]. Approximately 169,000 new female breast cancer patients are diagnosed annually, and this number has increased over the past forty years [2]. Henan Province has a large population with health outcomes at or below the national average [3]. The incidence of breast cancer in Henan Province is more than 35/100,000 [4]. While performance evaluations of medical service for breast cancer patients have the potential to improve clinical practices, the key factor is the selection of the evaluation tool itself [5].

Medical service performance evaluation tools are diverse and vary worldwide. For example, in Singapore, models based on the Singapore Quality Award criteria and the Balance Score Card (BSC) approach are used to evaluate the performance of hospitals [6]. The BSC method provides a framework that focuses on key management processes and evaluates the realization of the vision and strategy of a hospital based on the following four dimensions: finance, customer service, internal business, and innovation [7]. In America [8], the Joint Commission on Accreditation of Healthcare Organizations (JCAHO) implemented evidence-based standardized measures of performance in more than 3000 accredited hospitals. The measures were designed to track performance over time and compare hospitals based on the following six dimensions: safety, patient satisfaction, efficiency, clinical quality, financial management, and medical expenses [9]. In China, the primary tools used for performance management in hospitals focus on the following: financial management, human resource management, and clinical management [10]. Different evaluation methods are chosen according to the different evaluation objects, such as hospitals, departments and doctors [11]. For instance, the BSC method, key performance indicator (KPI) and achievement measurement method are used to evaluate the performance of hospitals, departments and doctors, respectively [5]. To achieve an ideal performance evaluation system, the most important feature is the accuracy of the evaluation results [12]. However, due to the inherent characteristics of medical services, including diversity, high risk, and difficulty performing comparisons, performance evaluations without risk adjustment, such as key performance indicators, cannot guarantee reliable results [13]. Therefore, an evaluation tool based on risk adjustment can improve the accuracy of the evaluation results.

The diagnosis related groups (DRGs) system was the first health management tool to group patients into clinically meaningful categories representing equivalent health resource usage. The DRGs system was first adopted in the state of New Jersey in 1980 [14] and was implemented by the US federal government as a payment system in 1983 [15]. Subsequently, several countries adopted the DRGs system [16]. Currently, this system is the most widely used risk adjustment tool [17]. Considering the importance of risk adjustment in performance evaluations, researchers at Peking University began to study the DRGs system as a performance evaluation tool in 2005 [12, 18, 19]. A series of medical service performance evaluation indicators were constructed to evaluate the range of available services for patients, service efficiency, and medical safety. The DRGs system has been shown to have several advantages over traditional evaluation methods. First, in contrast with the subjectivity of the scoring system used in the BSC [20], the DRGs evaluation indicators are based on objective data, such as the number of discharged patients, length of stay, medical cost and mortality. Therefore, an evaluation method using DRGs is likely to be more reliable and accurate than other methods. Second, the DRGs system effectively avoids biases in comparisons by adjusting case mixes across different hospitals [21]; thus, the results are more reliable and impartial [22, 23]. Third, this evaluation method is non-exclusive and can be combined with other performance evaluation methods [24]. Finally, continuous data are relatively easy to obtain because they are collected from medical records. Due to these advantages, medical service performance evaluation indicators conducted in Beijing are based on the DRGs system [12, 18, 19]. This line of studies has more recently been extended to other parts of China [25, 26], but to date, no study assessing the performance of DRGs-based medical service for breast cancer patients has been performed.

## Methods

### Data sources

Since 2012, medical institutions in Henan Province have adopted a common and uniform discharge abstract, commonly referred to as the front page of medical records (FPMR). The FPMR contains much information, including patient demographic information (age, sex, address, etc.), date of admission and discharge, diagnosis (principal diagnosis and other diagnoses), procedures (principal procedures and other procedures), hospitalization outcome, medical costs and prescription records. The diagnoses and procedures are coded according to the International Classification of Diseases, tenth revision (ICD-10) and International Classification of Diseases, Clinical Modification, 9th revision (ICD-9-CM-3), respectively.

In this study, FPMRs from all hospitals above class II that admitted breast cancer patients from Henan Province between 2016 and 2019 were reviewed. The relevant information of each case was carefully collated and assessed. Cases were included if they met the following criteria: (i) the ICD-10 code of the principal diagnosis contained C50: malignant neoplasm of the breast, and (ii) the date of discharge was between January 2016 and December 2019. Cases were excluded if they met the following criteria: (i) the length of stay was longer than 60 days, and (ii) critical information, such as the patient’s age, sex, diagnoses, procedures performed, discharge date, medical costs, or length of stay, was missing. Based on these criteria, we collected 103,760 records between January 2016 and December 2019.

### DRGs selection method

**Fig. 1:**
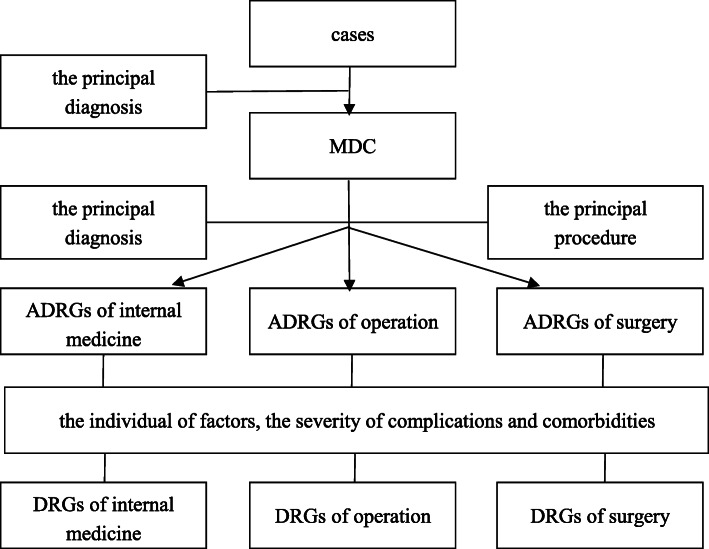
CN-DRGs grouping path

In 1988, the Institute of Hospital Management in Beijing took the lead in DRGs research. Subsequently, China developed its own DRGs system, laying the foundation for DRGs-based technology. In 2004, the Beijing DRGs project team introduced trial versions of Beijing DRGs (BJ-DRGs) based on studies conducted in the US and Australia. Because DRGs specific to Henan Province have not been developed, our study used CN-DRGs (2018 edition) as a risk adjustment tool. According to the CN-DRGs, 26 major diagnostic categories (MDCs) were established based on the primary diagnosis, and 806 DRGs were formed according to the patients’ individual factors (e.g., age, sex, etc.) and the severity of complications and comorbidities after the division of the adjacent DRGs (ADRGs) into surgery, internal medicine, and operations [27]. Figure 1 shows the grouping path of the CN-DRGs.

### DRG evaluation indicators

According to the studies conducted by Jian W [12, 18, 19], we constructed six objective medical service performance indicators to evaluate the available range of medical services, efficiency, and safety. The average level of the DRGs indicators of the hospitals included in the BJ-DRGs evaluation was selected as the standard during the calculation of the DRG indicators (Table 1).

**Table 1:** Health system performance evaluation indicators based on DRGs

Dimension	Indicators	Evaluation contents
**Availability**	Number of DRGs Total weight	The range of services available Total output of in-patient services
	Case-Mix Index (CMI)	Average technical difficulty level of treating diseases in each discipline
**Efficiency**	Charge Efficiency Index (CEI)	Cost of treating similar diseases
	Time Efficiency index (TEI)	Time for treating similar diseases
**Safety**	Inpatient mortality of low-risk group cases (IMLRG)	Mortality of diseases that are extremely unlikely to cause death

#### Service availability indicators [19]

Service availability could be evaluated by calculating the number of DRGs, total weight, and the case mix index (CMI). Together, these indicators captured the range of services available, the total output of medical services and the technical difficulty in treating patients after adjusting for each hospital case mix. The calculation methods for total weight and CMI are as follows:

$$ \mathrm{Total}\ \mathrm{weight}=\sum \mathrm{Each}\ \mathrm{DRG}\ \mathrm{weight}\times \mathrm{Number}\ \mathrm{of}\ \mathrm{cases}\ \mathrm{in}\ \mathrm{each}\ \mathrm{DRG} $$

$$ \mathrm{CMI}=\frac{\mathrm{Total}\ \mathrm{weight}}{\mathrm{Number}\ \mathrm{of}\ \mathrm{cases}\ \mathrm{in}\ \mathrm{Henan}} $$

The each DRG weight is the weight according to the DRG in the BJ-DRGs, which was calculated by dividing the average cost of each DRG group by the average cost of all cases in the Beijing region. CMI was the weighted average of each DRG weight.

#### Service efficiency indicators [12, 19]

Service efficiency was reflected by the following two indices: charge efficiency index (CEI) and time efficiency index (TEI). The two indicators are relative values that can be used to capture the cost and length of stay compared with the average level of the DRG indicators of the same diseases in the hospitals included in the BJ-DRGs evaluation. The larger the two values are, the lower the health service efficiency. If the TEI and CEI were greater than 1, the time efficiency and cost efficiency required to treat the same diseases were lower than those in the standard sample. The calculation methods for CEI and TEI are as follows.

$$ \mathrm{Cost}\ \mathrm{ratio}\left({k}^c\right)=\frac{\mathrm{average}\ \mathrm{cost}\ \mathrm{of}\ \mathrm{a}\ \mathrm{DRG}\ \mathrm{in}\ \mathrm{Hennan}\ \mathrm{Province}\left({c}_i\right)}{\mathrm{average}\ \mathrm{cost}\ \mathrm{of}\ \mathrm{a}\ \mathrm{DRG}\ \mathrm{in}\ \mathrm{BJ}-\mathrm{DRGs}\left(\overline{C_i}\right)} $$

$$ \mathrm{Average}\ \mathrm{length}\ \mathrm{of}\ \mathrm{stay}\ \left(\mathrm{ALOS}\right)\ \mathrm{ratio}\ \left({k}^l\right)=\frac{\mathrm{average}\ \mathrm{length}\ \mathrm{of}\ \mathrm{a}\ \mathrm{DRG}\ \mathrm{in}\ \mathrm{Hennan}\ \mathrm{Province}\left({l}_i\right)}{\mathrm{average}\ \mathrm{length}\ \mathrm{of}\ \mathrm{a}\ \mathrm{DRG}\ \mathrm{in}\ \mathrm{BJ}-\mathrm{DRGs}\left(\overline{L_i}\right)} $$

$$ \mathrm{CEI}=\frac{\sum \limits_j{k}_j^c{n}_j}{\sum \limits_j{n}_j} $$

$$ \mathrm{TEI}=\frac{\sum \limits_j{k}_j^{\mathrm{l}}{n}_j}{\sum \limits_j{n}_j} $$

where *n*_*j*_ represents the number of cases in DRG_*j*_. TEI and CEI are the weighted averages of *k*^*c*^ and *k*^*l*^, respectively.

#### Medical safety indicators

Inpatient mortality of low-risk group cases (IMLRP) was adopted to reflect medical safety. Different from traditional mortality indicators, IMLRP represents the mortality rate of diseases that are extremely unlikely to cause death and could be employed as an index of service quality [28]*.* Based on the experience of previous researchers [12, 18, 19], the procedure used to assign patients to lower risks levels was as follows [12, 18, 19]: (1) the mortality rate (*Mi*) of each DRG was calculated; (2) the natural logarithm of these mortality rates (*Ln (Mi)*) was computed in order to follow a normal distribution, the mean and standard deviation of *Ln (Mi)* were derived; and (3) IMLRP was defined as the mortality rate of the DRGs in which *Ln (Mi)* was less than one standard deviation below the mean value of *Ln (Mi)*.

### Statistical analysis

Continuous variables were expressed as the mean ± standard deviation. Ratios or rates were used to describe categorical variables. One-way analysis of variance was adopted to compare the differences in continuous variables among multiple groups. The chi-square test or Fisher’s exact probability was used to compare the differences in ratios or rates among different groups. The statistical significance was set a priori at *P* ≤ 0.05. Line charts were adopted to describe the time trend of continuous variables. All analyses were conducted in SPSS version 22.0.

## Results

### Sample characteristics

We collected 103,760 records of breast cancer patients between January 2016 and December 2019. There were 19,329 cases in 2016, 23,056 cases in 2017, 26,807 cases in 2018, and 34,568 cases in 2019, and the average annual growth rate of the number of cases was 21.38%. The number of cases rapidly increased during the study period. The mean patient ages from 2016 to 2019 were 50.77 ± 11.40, 51.12 ± 11.78, 51.40 ± 10.74 and 51.46 ± 10.70 years, respectively. In total, 46,936 cases (45.24%), 44,450 cases (42.84%) and 12,374 cases (11.92%) were admitted via the emergency department, outpatient department and other pathways, respectively. There were significant differences in mean patient age (*F* = 19.360, *P* = 0.000) and the distribution of admission routes (*x*^2^ = 1292.567, *P* = 0.000) among different years.

### Medical service availability

**Fig. 2:**
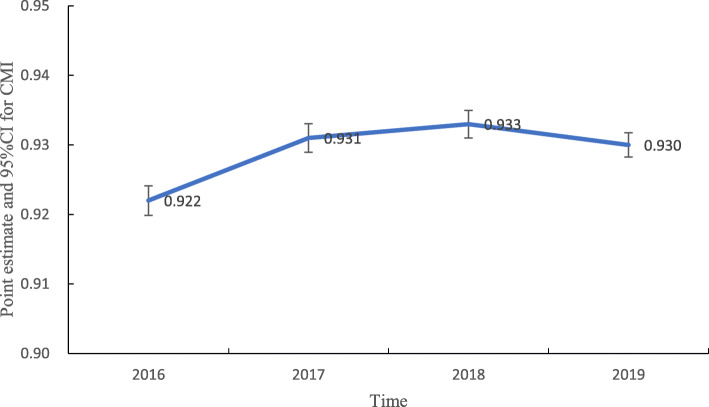
Trend of point estimates and 95%CIs for CMI in 2016 ~ 2019

In total, 22 DRGs comprised 17 surgical groups treating 35,313 patients and five internal medicine groups treating 68,447 patients. During the study period, the number of separate DRGs recorded was 18 in 2016, 17 in 2017, 19 in 2018, and 18 in 2019, and the distribution of the number of cases in separate DRGs was significantly different among different years (*x*^2^ = 432.419, *P* = 0.0000). The total number of weighted cases as represented by total weight were 17,821.58, 21,474.45, 25,021.86, and 32,132.81 in 2016, 2017, 2018 and 2019, respectively, and the average annual growth rate was 21.71% (Table 2). The CMI increased marginally from 2016 to 2019, and there were significant differences in CMI among groups (*F* = 20.344, *P* = 0.000). The point estimates and 95% confidence intervals (95%CIs) for CMI in 2016 ~ 2019 are shown in Fig. 2.

**Table 2:** Medical service availability in breast cancer patients between 2016 and 2019

Year	Number of cases	Number of DRGs	Total weight
**2016**	19,329	18	17,821.58
**2017**	23,056	17	21,474.45
**2018**	26,807	19	25,021.86
**2019**	34,568	18	32,132.81

**Fig. 3:**
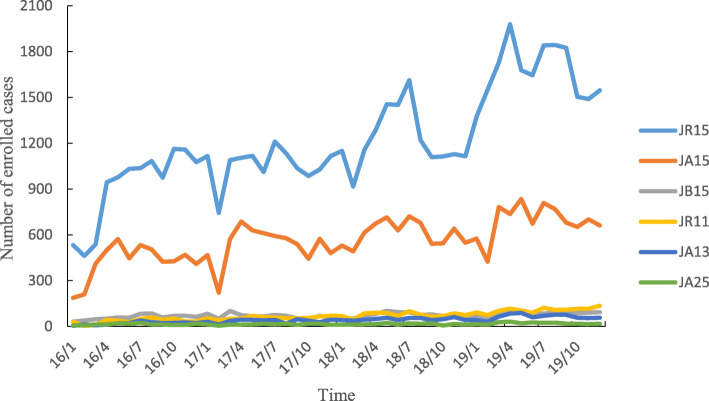
Number of enrolled cases of major DRGs varies monthly

**Fig. 4:**
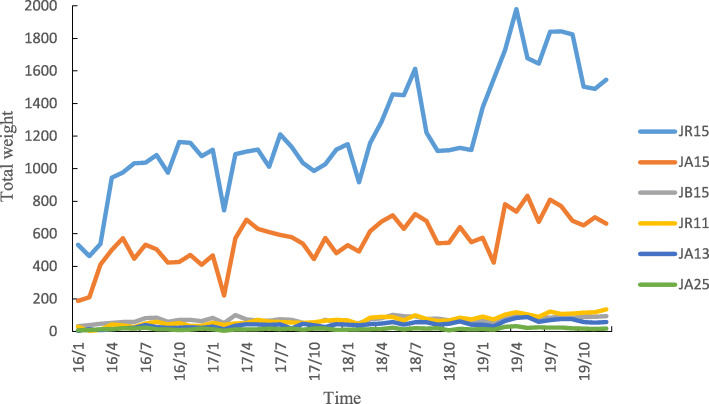
Total number of weight cases of major DRGs varies monthly

Six DRGs were associated with the overwhelming majority of all cases during the study period. Each of these six DRGs was associated with more than 1000 cases during the entire study period. Specifically, these six DRGs comprised JR15 (breast malignancy without complications or comorbidities, such as breast cancer patients who were admitted to the hospital for the first time who accepted nonsurgical treatment, including chemotherapy and radiation), JA15 (total mastectomy without complications or comorbidities, such as patients who underwent bilateral complete mastectomy without complications), JB15 (mammoplasty or other surgery without complications or comorbidities), JR11 (breast malignancy with severe complications or comorbidities), JA13 (total mastectomy with general complications or comorbidities), and JA25 (subtotal mastectomy without complications or comorbidities). The number of enrolled cases and the total weight of all six DRGs increased yearly, and the average annual growth rate of the total weight was 46.93% for JA13, 45.32% for JR11, 22.14% for JR15, 17.64% for JA15, 14.51% for JA25, and 12.98% for JB15 (Table 3). Throughout the study, the number of enrolled cases and total weight were always smaller during the first quarter of each year in all six DRGs (Figs. 3 and 4).

**Table 3:** Medical service availability of major DRGs between 2016 and 2019

DRGs	2016		2017		2018		2019	
	N(%)	Total weight	N(%)	Total weight	N(%)	Total weight	N(%)	Total weight
**JR15**	12,478 (64.56)	10,980.64	14,425 (62.57)	12,694.00	16,725 (62.39)	14,718.00	22,735 (65.77)	20,006.80
**JA15**	4719 (24.41)	5096.52	5923 (25.69)	6396.84	6787 (25.32)	7329.96	7682 (22.22)	8296.56
**JB15**	1106 (5.72)	718.90	1254 (5.44)	815.10	1406 (5.24)	913.90	1595 (4.61)	1036.75
**JR11**	261 (1.35)	420.21	426 (1.85)	685.86	569 (2.12)	916.09	801 (2.32)	1289.61
**JA13**	209 (1.08)	240.35	371 (1.61)	426.65	503 (1.88)	578.45	663 (1.92)	762.45
**JA25**	301 (1.56)	168.56	293 (1.27)	164.08	328 (1.22)	183.68	452 (1.31)	253.12

### Medical service efficiency

**Fig. 5:**
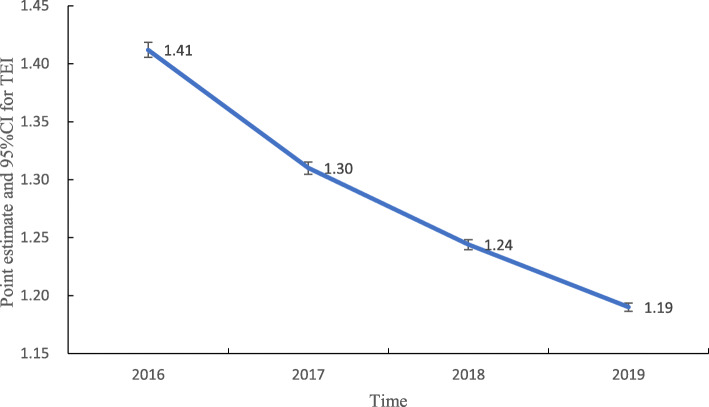
Trend of point estimates and 95%CIs for TEI in 2016 ~ 2019

**Fig. 6:**
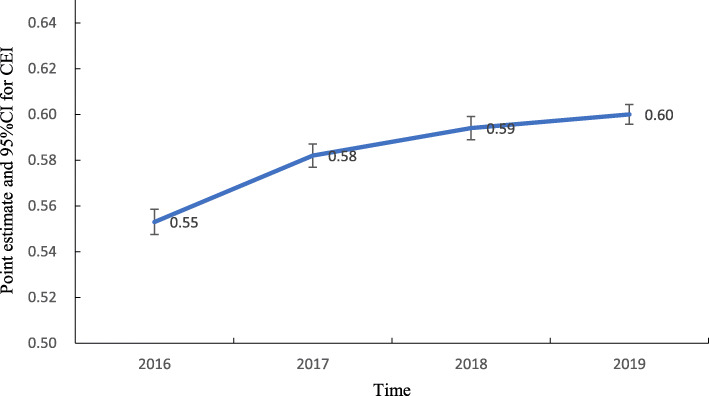
Trend of point estimates and 95%CIs for CEI in 2016 ~ 2019

The ALOS of breast cancer patients from 2016 to 2019 was 17.10, 15.40, 14.30, and 13.20, respectively. TEI decreased yearly, and in 2019, the TEI was 15.60% lower than that in 2016. There were significant differences in TEI among the four years (*F* = 224.876, *P* = 0.000). The average cost of breast cancer patients from 2016 to 2019 was ¥9875.85, ¥10,661.75, ¥11,077.87, and ¥11,102.41, respectively. CEI exhibited an increasing trend, and statistically significant differences were found in CEI among different years (*F* = 12.774, *P* = 0.000), but the average annual increase rate was small (2.94%). The point estimates and 95%CIs for TEI and CEI in 2016 ~ 2019 are shown in Figs. 5 and 6.

**Fig. 7:**
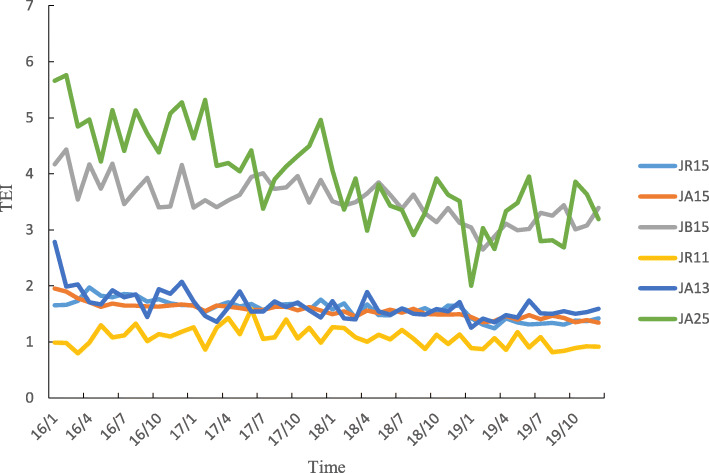
TEI of major DRGs varies monthly

**Fig. 8:**
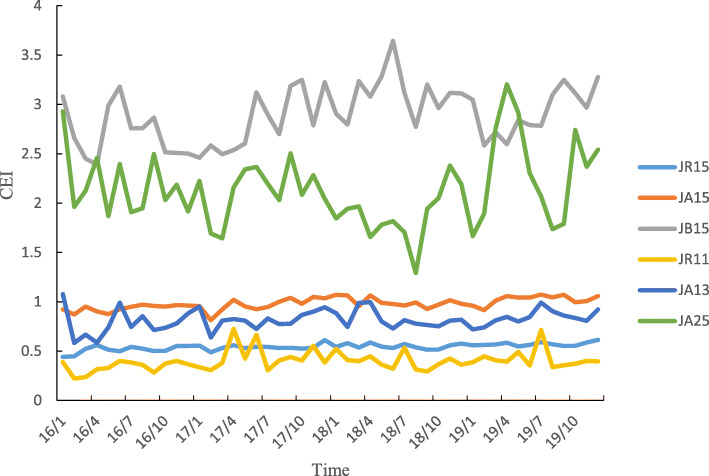
CEI of major DRGs varies monthly

Among the six DRGs, the TEI of JA25 was the highest, and the TEI of JR11 was the lowest. The TEIs of the six DRGs decreased yearly, and the TEI of JA25 decreased the most (31.15% lower in 2019 than in 2016). The CEI of JB15 was the highest, and the CEI of JR11 was the lowest. The CEIs of JR15, JA15 and JB15 increased slowly over the years, while the trends of the CEIs of the other three DRGs were not obvious (Table 4). During the same year, the temporal trends of the TEI and CEI of each DRG were inconsistent (Figs. 7 and 8).

**Table 4:** Medical service efficiency of major DRGs between 2016 and 2019

DRGs	2016		2017		2018		2019	
	TEI(95%CI)	CEI(95% CI)	TEI(95% CI)	CEI(95% CI)	TEI(95% CI)	CEI(95% CI)	TEI(95% CI)	CEI(95% CI)
**JR15**	1.77 (1.74,1.80)	0.52 (0.51,0.53)	1.65 (1.62,1.67)	0.54 (0.53,0.55)	1.57 (1.54,1.59)	0.55 (0.54,0.56)	1.50 (1.48,1.51)	0.57 (0.56,0.58)
**JA15**	1.69 (1.67,1.70)	0.94 (0.92,0.95)	1.61 (1.59,1.62)	0.98 (0.96,0.99)	1.52 (1.50,1.54)	0.99 (0.98,1.01)	1.50 (1.48,1.51)	1.03 (1.02,1.04)
**JB15**	3.80 (3.69,3.91)	2.71 (2.60,2.82)	3.66 (3.56,3.76)	2.78 (2.68,2.88)	3.48 (3.39,3.57)	3.13 (3.03,3.24)	3.33 (3.25,3.41)	2.93 (2.83,3.02)
**JR11**	1.13 (1.02,1.24)	0.35 (0.32,0.39)	1.20 (1.11,1.29)	0.45 (0.40,0.50)	1.09 (1.02,1.17)	0.40 (0.36,0.44)	1.03 (0.97,1.09)	0.43 (0.38,0.47)
**JA13**	1.84 (1.74,1.95)	0.78 (0.71,0.84)	1.60 (1.54,1.67)	0.83 (0.78,0.88)	1.58 (1.51,1.64)	0.82 (0.78,0.87)	1.57 (1.52,1.63)	0.85 (0.81,0.89)
**JA25**	4.88 (4.65,5.12)	2.13 (2.00,2.27)	4.26 (4.04,4.29)	2.16 (1.98,2.34)	3.48 (3.27,3.69)	1.85 (1.72,1.99)	3.36 (3.19,3.54)	2.39 (2.21,2.58)

### Medical service safety

**Fig. 9:**
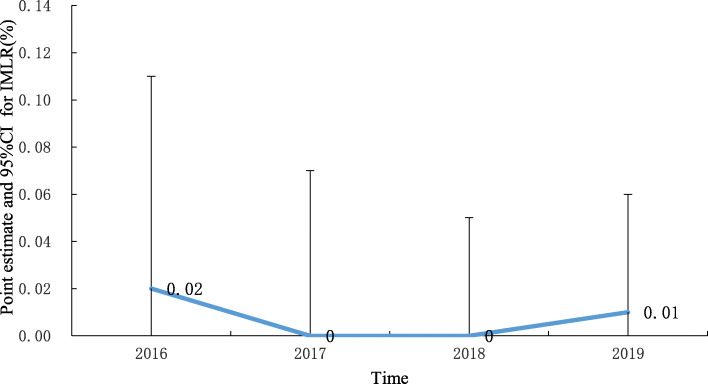
Trend of point estimates and 95%CIs for IMLRP in 2016 ~ 2019

The mortality rate of breast cancer patients was 0.41, 0.36, 0.43 and 0.36% in 2016, 2017, 2018 and 2019, respectively, while two deaths occurred among the low-risk group cases (one in 2016 and another in 2019), and both cases occurred in the JA15 DRG. IMLRP was 0.02, 0, 0 and 0.01% in 2016, 2017, 2018 and 2019, respectively and there were no significant differences for IMLRP of the four years (*P* = 0.6684). The point estimates and 95%CIs for IMLRP in 2016 ~ 2019 are shown in Fig. 9.

## Discussion

DRGs is a management system that classifies patients into several diagnostic groups based on disease diagnosis, treatment, complications, age, outcome and other factors. Using the principles of clinical and resource-utilization similarity [29], we identified 103,760 cases of breast cancer that were divided into 22 DRGs during the study period. The number of separate DRGs recorded during the study period varied (18 in 2016, 17 in 2017, 19 in 2018, and 18 in 2019). Because the mean patient age, diseases and DRGs were inconsistent in different years, the number of discharged patients, length of stay, medical costs and other conventional indicators without risk adjustment were not directly comparable across different years. We effectively avoided biases due to the heterogeneity of diseases and DRGs by assigning different weights to different DRGs, thereby allowing for comparisons to be performed across different years. In addition, the adoption of the average level of DRGs indicators in hospitals in Beijing as benchmark data was beneficial for comparisons between Henan Province and developed regions.

We adopted the CN-DRGs (2018 edition) grouping method to analyse the performance of medical service in patients with breast cancer. This analysis showed that the medical service performance improved during the study period, particularly in the total number of weighted cases, as represented by total weight, and the length of stay of those cases, as represented by the TEI. The total number of weighted cases increased yearly, and the CMI increased marginally. The number and total weight of the enrolled cases in the six DRGs (JR15, JA15, JB15, JR11, JA13, and JA25) also increased yearly. JR11 (breast malignancy with severe complications or comorbidities) and JA13 (total mastectomy with general complications or comorbidities) were the two fastest-growing groups. This finding may be related to the increase in the demand for medical services, improvements in the supply capacity of health services, advancement of medical insurance policies, and nullification of medicine markups. First, the incidence of breast cancer among residents in Henan Province has been increasing in recent years [30*,* 31], and the demand for health services for breast cancer has increased, which may increase the hospitalization rate of breast cancer year by year. Second, the introduction of policies, such as the regional medical union, “County-level Clinical Key Specialty Project” and “the 515 Action Plan”, increased financial support for medical institutions and improved access to primary health care institutions. Third, the implementation of a series of medical insurance policies reduces the burden of the patients’ medical treatment cost and may increase the rate that patients seek a doctor’s advice. In 2016, Henan Province announced the decision to include the treatment of breast cancer in the first batch of critical illness insurance, and breast cancer patients could receive additional medical insurance reimbursements after basic medical insurance reimbursements [32]. In 2017, Henan Province announced that all people of low economic status could obtain supplementary medical insurance reimbursements after basic medical insurance and critical illness insurance reimbursements [33]. Finally, public hospitals in the capital of Henan Province nullified the policy of medicine markups in 2017, which reduced the cost of drugs and may increase the rate that patients seek a doctor’s advice [34].

The TEI of all enrolled breast cancer patients was greater than 1 between 2016 and 2019 but decreased yearly, with the largest decrease observed in JA25 (subtotal mastectomy without complications or comorbidities). This finding indicates that for similar disease, the time efficiency of medical institutions in Henan Province was worse than that of institutions in Beijing, but this measure improved yearly, particularly for patients who underwent surgery. This finding may be associated with the implementation of clinical pathways [35, 36]. The CEI of all enrolled breast cancer patients was less than 1 between 2016 and 2019, while that of JR15 (breast malignancy without complications or comorbidities), JA15 (total mastectomy without complications or comorbidities), and JB15 (mammoplasty or other surgery without complications or comorbidities) increased slowly year to year from 2016 to 2019. These results indicated that the cost efficiency of medical institutions in Henan Province was better than that of institutions in Beijing, but the cost of treating certain diseases slightly increased over time. The implementation of endoscopic-assisted modified radical mastectomy through a ring areola incision and the introduction of advanced equipment may explain the increase in cost. The abovementioned results show that the efficiency of medical services in Henan Province has improved, but active measures must be taken to continuously support ongoing improvements in efficiency. Effective countermeasures can rationally shorten the average length of stay. Hospitals should actively learn advanced methods, establish monitoring targets, shorten examination and treatment waiting times, optimize processes, improve the efficiency of medical-technical departments, implement clinical pathways and single disease management, and effectively control nosocomial infections [37, 38]. In addition, hospitals should pay attention to strictly controlling costs and reducing medical expenses for patients while using advanced technology and introducing advanced equipment to maintain high-quality medical services [39].

The mortality rate of breast cancer patients was 0.41, 0.36, 0.43 and 0.36% in 2016, 2017, 2018 and 2019, and the annual IMLRP was 0.02, 0, 0 and 0.01% in 2016, 2017, 2018 and 2019, respectively. Although there were no significant differences for IMLRP of the four years, there were still two deaths among the low-risk group cases. These results suggested that medical staff should continue to strengthen their medical safety precautions. The PDCA cycle can be adopted to achieve continuous improvements in medical quality, and hospitals should promote analyses of cases of low-risk death to discover their own problems in the diagnosis and treatment process and address these problems in a targeted manner [40].

### Study limitations

Several potential limitations should be noted. First, the grouping methods and DRGs evaluation indicators system have not been customized for clinical practice in Henan Province. Therefore, we used the relatively mature performance evaluation system of DRGs implemented in Beijing; nonetheless, the suitability of this system should be verified. Second, highly accurate FPMRs are required for the performance evaluation of medical service based on DRGs. The FPMR data used in our study were provided by medical institutions. Although the relevant information of each case was carefully collated and assessed, the quality of disease and procedure coding in the FPMR was difficult to control and may have affected the accuracy of the evaluation results. To better perform DRGs-based medical service performance evaluation, it is important to accelerate the design of a localized scheme for DRGs to create a DRGs grouping scheme and evaluation indicators that are highly customized to the unique circumstances of Henan Province. Simultaneously, it is crucial to strengthen personnel training in medical institutions to improve the quality and coding accuracy in the FPMR. Third, there are many indicators that can reflect medical safety. The use of mortality in the low-risk group to reflect medical safety may not be very comprehensive, and further research is needed to improve upon this aspect.

## Conclusions

DRGs represent a risk adjustment tool that can be used to more impartially compare the performance of a health system across different years. Between 2016 and 2019, the total number of weighted cases, as represented by total weight, increased from year to year, indicating that medical service availability in Henan Province continuously improved. The TEI was greater than 1 but decreased yearly, while the CEI was less than 1 but slightly increased between 2016 and 2019, indicating that medical efficiency improved but still needs greater improvement. Two deaths occurred among the low-risk group cases, illustrating that there are opportunities for greater improvement in medical safety.

## Data Availability

The datasets generated and/or analysed during our study are not publicly available because Health Information Center of Henan Province required all researchers not to disclose the database. It may be possible for other researchers to require access to the dataset directly from the Health Information Center of Henan Province.

## Abbreviations

DRGs: Diagnosis related groups
FPMR: The front pages of medical records
CN-DRGs: China DRGs
CMI: Case-mix Index
CEI: Charge efficiency index
TEI: Time efficiency index
IMLRG: Inpatient mortality for low-risk group cases
BSC: Balance Score Card
JCAHO: the Joint Commission on Accreditation of Healthcare Organizations
KPI: Key Performance Indicators
ICD-10: the International Classification of Diseases, Tenth Revision
ICD-9-CM-3: International Classification of Diseases, Clinical Modification, 9th revision
BJ-DRGs: Beijing DRGs
MDCs: Major diagnostic categories
ADRGs: Adjacent DRGs
95%CIs: 95% confidence intervals
